# Adaptation to Endoplasmic Reticulum Stress in *Candida albicans* Relies on the Activity of the Hog1 Mitogen-Activated Protein Kinase

**DOI:** 10.3389/fmicb.2021.794855

**Published:** 2022-01-06

**Authors:** Farha Husain, Prerna Pathak, Elvira Román, Jesús Pla, Sneh Lata Panwar

**Affiliations:** ^1^Yeast Molecular Genetics Laboratory, School of Life Sciences, Jawaharlal Nehru University, New Delhi, India; ^2^Departamento de Microbiología y Parasitología-IRYCIS, Facultad de Farmacia, Universidad Complutense de Madrid, Madrid, Spain

**Keywords:** *Candida*, tunicamycin, endoplasmic reticulum stress, unfolded protein response, HOG MAPK, Ire1, Hac1

## Abstract

Adaptation to ER stress is linked to the pathogenicity of *C. albicans*. The fungus responds to ER stress primarily by activating the conserved Ire1-Hac1-dependent unfolded protein response (UPR) pathway. Subsequently, when ER homeostasis is re-established, the UPR is attenuated in a timely manner, a facet that is unexplored in *C. albicans*. Here, we show that *C. albicans* licenses the HOG (high-osmolarity glycerol) MAPK pathway for abating ER stress as evidenced by activation and translocation of Hog1 to the nucleus during tunicamycin-induced ER stress. We find that, once activated, Hog1 attenuates the activity of Ire1-dependent UPR, thus facilitating adaptation to ER stress. We use the previously established assay, where the disappearance of the UPR-induced spliced *HAC1* mRNA correlates with the re-establishment of ER homeostasis, to investigate attenuation of the UPR in *C. albicans*. *hog1*Δ/Δ cells retain spliced *HAC1* mRNA levels for longer duration reflecting the delay in attenuating Ire1-dependent UPR. Conversely, compromising the expression of Ire1 (*ire1* DX mutant strain) results in diminished levels of phosphorylated Hog1, restating the cross-talk between Ire1 and HOG pathways. Phosphorylation signal to Hog1 MAP kinase is relayed through Ssk1 in response to ER stress as inactivation of Ssk1 abrogates Hog1 phosphorylation in *C. albicans*. Additionally, Hog1 depends on its cytosolic as well as nuclear activity for mediating ER stress-specific responses in the fungus. Our results show that HOG pathway serves as a point of cross-talk with the UPR pathway, thus extending the role of this signaling pathway in promoting adaptation to ER stress in *C. albicans*. Additionally, this study integrates this MAPK pathway into the little known frame of ER stress adaptation pathways in *C. albicans*.

## Introduction

The endoplasmic reticulum (ER), in all eukaryotes, facilitates the efficient folding and transport of secretory and membrane proteins to their targeted locations (Mori et al., 1993). Protein biosynthesis in the ER is guided by chaperones and folding enzymes in a fail-safe manner by quality control systems. However, the increased demand for secretion during environmental or developmental changes overwhelms the protein folding machinery within the ER. Consequentially, the probability of unwarranted interactions during protein folding is enhanced, resulting in the accumulation of misfolded proteins in this organelle. This imbalance in the protein-folding homeostasis, referred to as ER stress, activates adaptive signaling pathways to alleviate the stress. The evolutionary conserved unfolded protein response (UPR) pathway plays a key role in adapting to ER stress in *Saccharomyces cerevisiae* and pathogenic fungi (Krishnan and Askew, 2014).

Pathogenic fungi including *C. albicans* encounter ER stress during infection in a mammalian host. *C. albicans*, as a harmless commensal, resides in the gastrointestinal tract (GI), mouth, skin, and female reproductive tract of healthy individuals (Kennedy and Volz, 1985; Achkar and Fries, 2010; Ghannoum et al., 2010; Kumamoto, 2011; Calderone and Clancy, 2012; Kan et al., 2020). The benign commensal turns into a pathogen, causing life-threatening invasive and systemic candidiasis, when the host develops immune deficiency, epithelial damage, or microbiota dysbiosis (Edmond et al., 1999; Perlroth et al., 2007; Mayer et al., 2013; Bongomin et al., 2017). For successful infection, this fungus activates the ER stress response pathways to strike a balance between adaptation to ER stress and ER stress-induced cell death. The ER stress-induced UPR pathway in *C. albicans* is comprised of the conserved ER-resident transmembrane protein kinase Ire1 and its downstream transcription factor Hac1 (Sircaik et al., 2021). In *S. cerevisiae*, binding of misfolded proteins to the ER lumenal domain of Ire1 triggers its autophosphorylation and oligomerization, and activates ribonuclease (RNase) activity. Ire1 through its RNase activity excises the 252-bp and 19-bp intron from *S. cerevisiae* and *C. albicans HAC1* mRNA, respectively, thus generating Hac1 protein (Cox et al., 1993; Kohno et al., 1993; Wimalasena et al., 2008; Korennykh et al., 2009; Sircaik et al., 2021). Thereafter, Hac1 coordinates the restoration of ER homeostasis by initiating the expression of proteins involved in protein folding and secretion and regulating the disposal of misfolded proteins by ER-associated degradation (ERAD) (Wimalasena et al., 2008; Ruggiano et al., 2014; Chang et al., 2018). In yeast, once protein-folding homeostasis is re-established, the Ire1-dependent UPR activity terminates, signaling recovery from the stress (Chawla et al., 2011; Chang et al., 2018). Sustained expression of the ER stress-induced spliced *HAC1* mRNA and overexpression of Ire1 results in cell death in *S. cerevisiae*, reflecting the importance of timely termination of the UPR (Kawahara et al., 1997).

In *C. albicans*, while events leading to the activation of the Ire1-dependent UPR have been established (Sircaik et al., 2021), steps that mediate its timely attenuation remain uninvestigated. High-osmolarity glycerol (HOG) MAP kinase pathway promotes recovery from ER stress by mediating attenuation of the Ire1-dependent UPR and regulating the expression of a specific subset of genes (*HSP12*, *YMR103C*, and *YPL088W*) during late stage of ER stress response in *S. cerevisiae.* Induction of autophagy, considered as one of the downstream events during ER stress in yeast, is also regulated by Hog1 in the budding yeast. Hog1, by regulating the stability of Atg8, a critical autophagy protein, contributes to the activation of autophagy. Therefore, Hog1 activation facilitates modulation of multiple cellular processes specifically during the late stage of ER stress, resulting in counteracting the stress in *S. cerevisiae* (Bicknell et al., 2010). In *C. albicans*, Hog1 is activated by phosphorylation in response to osmotic and oxidative stresses (Alonso-Monge et al., 2003; Smith et al., 2004), regulates virulence traits (Munro et al., 2007; Su et al., 2013; Hopke et al., 2016), and is crucial for the survival of the fungus within the host (Alonso-Monge et al., 1999; Arana et al., 2007; Prieto et al., 2014). Subsequent to osmostress-induced phosphorylation, Hog1 translocates to the nucleus (Arana et al., 2005; Kaloriti et al., 2014) where it activates the transcriptional program that promotes adaptation to various stresses (Enjalbert et al., 2006). Dephosphorylated Hog1 migrates back to the cytoplasm where it is required for maintaining signal fidelity and impeding cross-talk to other MAPK pathways (Arana et al., 2005; Eisman et al., 2006; Cheetham et al., 2011). Despite the central role of Hog1 in handling multiple stresses, its role during ER stress remains unaddressed in *C. albicans*.

Here, we explore the role of *C. albicans* Hog1 and bring forward the mode of action of this MAP kinase during tunicamycin-induced ER stress. We show that Hog1 is phosphorylated in Ssk1-dependent manner and migrates to the nucleus specifically during the late stage of ER stress response. For mediating ER stress resistance, Hog1 is dependent not only on its nuclear activity but also requires its cytosolic activity, thus highlighting the role of Hog1-dependent cytosolic proteins during ER stress in *C. albicans*. Moreover, once activated, HOG MAPK pathway promotes adaptation to ER stress by facilitating the timely attenuation of the UPR in *C. albicans*. All told, the finding that HOG pathway abates ER stress by orchestrating the Ire1-dependent UPR uncovers a new role for this MAP kinase in the pathogenic fungus.

## Materials and Methods

### Strain, Chemicals, and Growth Conditions

The *C. albicans* strains used in this study are listed in Table 1. All strains were maintained as frozen stocks and propagated at 30°C in defined medium, YEPD (1% yeast extract, 2% peptone, and 2% glucose). To stimulate the UPR in *C. albicans*, stationary phase cells were grown to mid-exponential phase in YEPD at 30°C with shaking (220 rpm), containing tunicamycin at indicated concentration. All the supplements and chemicals, β-mercaptoethanol, tunicamycin, and Calcofluor white, were purchased from Sigma-Aldrich. Dithiothreitol was obtained from SRL (Sisco Research Laboratories Pvt. Ltd).

**TABLE 1:** Strains used in this study.

Parent	Genotype	Strain	Source of reference
SC5314		Wild type	Gillum et al., 1984
RM1000	*Ura3Δ*:*imm434/ura3DΔ*:*imm434* *his1Δ*:*hisG/his1Δ*:*hisG*	Wild type	Negredo et al., 1997
CNC15	*RM1000* *hog1*:*hisG/hog1*:*hisG*	*hog1*Δ/Δ	Arana et al., 2005
BRD4	*RM1000* *pbs2Δ*:*cat/pbs2Δ:cat*	*pbs2*Δ/Δ	Arana et al., 2005
BRD8	*RM1000* *hog1*:*hisG/hog1*:*hisG pbs2Δ*:*cat/pbs2Δ* :cat	*hog1pbs2*Δ/Δ	Arana et al., 2005
hAHGI	*RM1000* *hog1*:*hisG/hog1*:*hisG ACT1p-HOG1 GFP*:*leu2/LEU2*	*HOG1*-GFP	Arana et al., 2005
BRD4G1	*pbs2Δ*:*cat/pbs2Δ*:*cat ACT1p-PBS2-GFP*:*leu2/LEU2*	*pbs2*Δ/Δ-*PBS2*	Arana et al., 2005
CSSK21	*ura3*:*imm434/ura3*:*imm434 ssk1*:*hisG/ssk1*:*hisG-URA3-hisG*	*ssk1*Δ/Δ	Arana et al., 2005
REP3	*ura3*:*imm434/ura3*:*imm434 his1*:*hisG/his1*:*hisG sho1*:*hisG/sho1*:*hisG-URA3-hisG*	*sho1*Δ/Δ	Arana et al., 2005
CW906	*ura3*Δ*iro1*Δ*:*λ*imm434arg4:hisG, his1*:*hisG ARG4:IRE1-DX1 ura3*Δ*iro1*Δ*:*λ*imm434arg4:hisGhis1*:*hisG*Δ*ire1:URA3*	*ire1* DX	Woolford et al., 2016
TW04	*ura3*:*kimm434/ura3*:*kimm434,his1*:*hisG/h is1*:*hisG, arg4*:*hisG/arg4*:*hisG, hac1:*loxP*/hac1*:*loxP,CIp30*	*hac1*Δ/Δ	Wimalasena et al., 2008
FH1	*ura3*Δ*iro1*Δ:λimm434arg4:*hisG*, *his1*:*hisG* ARG4:*IRE*-DX1 ura3Δiro1Δ:λimm434arg4:hisGhis1:hisG Δ*ire1*:*URA3*, *ura3*:*imm434/ura3*:*imm434 ssk1*:*hisG/ssk1*:*hisG-URA3-his*	*ire1*DX*ssk1*Δ/Δ	This study
JC2171	(RM1000) *loxP*, + pACT1-HOG1-GFP-NLS *hog1*:*loxP-ura3-loxP*, *hog1*:*loxP-HIS1-loxP*, + *pACT1-HOG1-GFP-NLS*	Hog1-GFP-NLS	Day et al., 2017
JC2172	(RM1000) *loxP*, + pACT1-HOG1-GFP-CaaX *hog1*:*loxP-ura3-loxP*, *hog1*:*loxP-HIS1-loxP*, + *pACT1-HOG1-GFP-CaaX*	Hog1-GFP-CaaX	Day et al., 2017

### Strain Construction

A transient CRISPR system to target *SSK1* was used that consisted of separate *CaCAS9* and sgRNA expression cassettes. *CaCAS9* cassette was amplified by PCR from pV1093 with the pair of primers CaCas9/for and CaCas9/rev. We chose a gRNA 219 bp downstream from the *SSK1* start codon and the sgRNA was generated through three PCRs steps to fuse *SNR52* promoter (amplified in PCR1 with the primers SNR52/F and SNR/R_SSK1) and the sgRNA (amplified in PCR2 with primers sgRNA/F_SSK1 and sgRNA/R). In the second round, primer extension was performed to fuse PCR1 and 2 and, finally, in the third step, PCR with nested primers SNR52/N and sgRNA/N was performed to generate the sgRNA expression cassette. An *ssk1*Δ:*NAT1* construct for the complete deletion of *SKK1* had 80-bp arms homologous to sequences upstream (89-bp from the start codon) and 9-bp immediately downstream the stop codon and the *NAT1* marker for selection. *ire1* DX mutant strain (Table 1) was transformed with *ssk1*Δ:*NAT1* construct and PCR products and nourseothricin resistant transformants were selected and analyzed for *SSK1* deletion by PCR genotyping. We checked for *SSK1* deletion (using primers Comp_SSK1_del_F and Comp_SSK1_del_R) and the absence of *SSK1* wild type gene (using primers Up-comp-SSK1 and Comp_SSK1_del_R). From six colonies analyzed in *ire1* background, 50% of them had both alleles deleted and 50% just one. Primers used in the study are listed in Supplementary Table 1.

### Drug Susceptibility and Viability Assays

*Candida albicans* strains were grown overnight on YEPD plates at 30°C. Cells were then diluted in 5 ml of 0.9% saline solution to OD_600_ of 0.1. Once the OD was set at 0.1, the culture was diluted 10 times with saline and subject to 5-fold serial dilutions. Then, 5 μl of culture from each of the dilutions was finally spotted on agar plates containing indicated drugs. The plates were incubated at 30°C and photographed after 48 h.

For viability assay, *Candida* strains were grown in YEPD medium overnight. The cells were resuspended in fresh YEPD medium to an OD_600_ of 0.3 and grown until OD_600_ of 1.0 at 30*^o^*C. Exponentially growing cells were collected by centrifugation and resuspended in YEPD containing 20 μg/ml tunicamycin and kept at 30^°^C. Samples were taken at different time points and spotted onto YEPD plates (10^5^ cells in 10 μl) and incubated for 24 h at 30°C.

### Protein Extraction and Immunoblot Analysis

For the Hog1 phosphorylation blot, overnight cultures were diluted in fresh YEPD media to an OD_600_ of 0.3 and grown until they reached an OD_600_ of 1.0 at 30*^o^*C. Lysates were prepared in ice-cold lysis buffer (150 mM NaCl, 1% Triton X-100, 0.1% SDS, 1 mM phenylmethylsulfonyl fluoride and protease inhibitor mixture) using acid-washed glass beads. The cell lysate was centrifuged at 4000 rpm for 2 min at room temperature, and the supernatant was used for further analysis. Protein concentration of lysates was estimated using BCA protein assay kit (BioBasic, SK3021). Then, 50 μg of the protein samples were separated by 10% sodium dodecyl sulfate-polyacrylamide (SDS-PAGE) gel electrophoresis. Protein was transferred to PVDF membrane (0.45 μm pore size; Merck-millipore) at 90 V for 2 to 3 h followed by blocking with 3% BSA (SRL) in Tris-Buffered saline (TBS) with 0.1% Tween-20 for 1 h. The blot was then probed with primary antibody 1:1000 for anti-Phospho Hog1 (phospho-p38 MAPK antibody, Thr180/Tyr182; #9215; Cell Signaling Technology) and 1:300 for anti-Hog1 (A-8: sc-16597, Santa Cruz Biotechnology) at 4°C overnight followed by washing thrice with TBST (TBS + 0.1% Tween-20). Secondary HRP-conjugated antibody (Goat rabbit IgG-HRP Sigma/Goat anti-mouse IgG-HRP; Sigma) was then added at a dilution of 1:5000 for 1 h, followed by three washes with TBST. The blot was developed on an X-ray film using ECL reagent (BioRad Clarity™ Western ECL substrate).

### Fluorescence Microscopy

Yeast strains were grown at 30°C in YEPD medium to an OD_600_ of 0.1. In the case of treated cells, NaCl or tunicamycin was added to the concentration as described and 1 ml samples were taken at different time points (30–180 min) after the addition of 2 μg/ml of tunicamycin or 5 min after exposure to 1 M NaCl (positive control). Samples were centrifuged at 6000 rpm for 2 min and washed twice with 1X PBS. Cells were fixed with 3.7% formaldehyde (kept at –20°C) for 5 min, centrifuged at 6000 rpm for 1 min, and washed twice with 1X PBS. DAPI (4′,6-diamidino-2-phenylindole) was added to a final concentration of 1 μg/ml to stain the DNA and incubated at least for 15 min. Digital images were captured by confocal using a 100× oil objective lens.

### RNA Extraction and Quantitative Real-Time PCR

*Candida albicans* strains were grown overnight in YEPD, subcultured from a starting OD_600_ of 0.3 in fresh YEPD and incubated at 30°C until OD_600_ reached 1.0. The desired drugs/compounds of interest were added to the media and culture was allowed to grow for indicated time periods. Cells were collected by centrifugation at 5000 rpm for 5 min at 4°C from treated and untreated control samples and total RNA was isolated using the RNeasy mini kit (Qiagen). Extracted RNA was treated with DN*ase* I (Thermo Scientific) to remove contaminating DNA and cDNA was synthesized with a RevertAid™ H Minus First Strand cDNA synthesis kit (Thermo Scientific) following the manufacturer’s protocol. All real-time polymerase chain reaction (PCR) reactions were performed in a volume of 25 μl using Thermo Scientific Maxima SYBR Green mix in a 96-well plate. For relative quantification of gene expression, the comparative C_*T*_ method was used. Fold change (treated/untreated) is calculated by 2^–ΔΔ^ *^C^*_*T*_, normalized to *ACT1* (endogenous control) with untreated wild type or mutant strains as calibrators. All values are mean ± S.D and are derived from three independent RNA preparations. The data were analyzed statistically using Student’s *t*-test. A *p* ≤ 0.05 was considered statistically significant. The qRT-PCR primers used in this study were designed by Primer Express 3.0 and are listed in Supplementary Table 2.

### Determination of *HAC1* mRNA Splicing

*Candida albicans* cells were treated with tunicamycin for different time points and total RNA was prepared by following the procedure described above. cDNA was prepared by using RevertAid™ H Minus First Strand cDNA synthesis kit (Thermo Scientific) and *HAC1* mRNA splicing was measured by the following:

(i) Reverse Transcriptase-PCR (RT-PCR) using *HAC1* gene-specific primers HAC1SP (F) and HAC1SP (R) (Supplementary Table 2) and the PCR product was analyzed on 4% agarose gel.

(ii) qRT-PCR was performed with TaqMan probe specific for spliced *HAC1* (*HAC1^s^*) or primers recognizing the unspliced *HAC1* (*HAC1^u^*) isoform. TaqMan Universal PCR Master Mix and primer-probe mixes were obtained from Applied Biosystems by Life Technologies. *ACT1* was used as the internal control and transcript level of the gene of interest was normalized to *ACT1* levels. Fold changes are means ± S.D and are derived from three independent RNA preparations.

### Glycerol Production Assay

*Candida* strains were grown at 30°C in YEPD medium to an OD_600_ of 1.0. Cells were collected by centrifugation and resuspended in YEPD containing 2 μg/ml tunicamycin or 1 M sorbitol (positive control) for 3 h. After 3 h, 1 ml of the culture was removed and pelleted. Then, 1 ml of Tris-Cl (0.1 M) was added and samples were boiled for 10 min at 90°C. Following, 10 μl of this sample was taken for assaying the glycerol content. Glycerol was determined colorimetrically with a commercial kit (Sigma) following the manufacturer’s instructions. The values obtained are expressed as μM of glycerol per mg of yeast cells, dry weight. For tunicamycin-treated samples, increase in glycerol content was estimated after subtraction of the values measured in the control assays containing DMSO alone. The values shown represent means ± S.D and are derived from the average of three independent experiments.

### Statistical Analysis

All the data were plotted and analyzed using GraphPad Software. Intergroup comparisons were made using the Student’s *t*-test. *P* value of ≤ 0.05 was considered significant.

## Results

### High-Osmolarity Glycerol Pathway Mediates Endoplasmic Reticulum Stress Resistance

**FIGURE 1:**
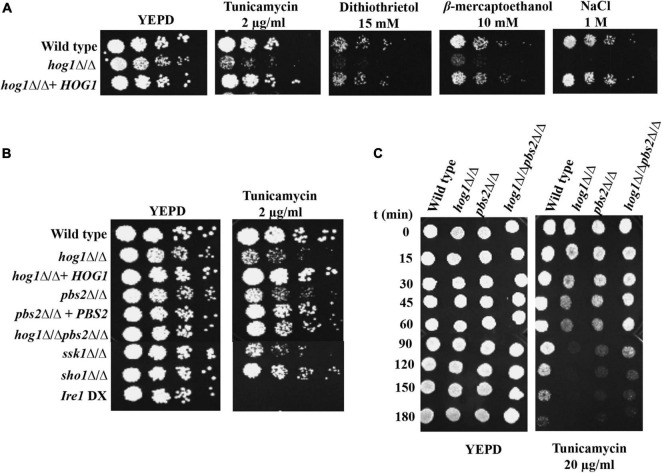
Growth of *hog1*Δ/Δ and mutants of upstream components of HOG MAPK pathway during ER stress. **(A)** and **(B)** Serial dilutions of cell suspensions of the indicated strains were spotted onto YEPD plates supplemented with drugs at indicated concentrations and incubated at 30°C for 48 h. **(C)** Exponentially growing cells were exposed to 20 μg/ml tunicamycin in liquid YEPD medium and kept at 30°C. Samples were taken at different times and spotted onto YEPD plates (10^5^ cells in 10 μl) and incubated for 24 h at 30°C.

Considering the role of HOG pathway during tunicamycin (Tm)-induced ER stress resistance in *S. cerevisiae* (Torres-Quiroz et al., 2010), we were interested in probing the role of the *C. albicans* HOG pathway during ER stress. To this end, we first tested the *hog1*Δ/Δ cells for growth in presence of well-known ER stressors such as Tm, a natural inhibitor of *N*-linked glycosylation, dithiothrietol (DTT), and β-mercaptoethanol, all of which lead to misfolding of proteins in the ER, thus resulting in ER stress. YEPD supplemented with 1M NaCl (osmostressor) was used as control because *hog1*Δ/Δ is known to exhibit increased susceptibility to this compound (San José et al., 1996). A conditional mutant strain, *ire1* DX (CW906; Table 1), that exhibits diminished expression of *IRE1* and is unable to grow in presence of Tm in *C. albicans* (Woolford et al., 2016; Sircaik et al., 2021) was also used as a control in the spot assays. As observed in Figure 1A, *hog1*Δ/Δ cells exhibited increased susceptibility to all the ER stressors, compared to the wild type and the reconstituted strain (Figure 1A). We also included *pbs2*Δ/Δ, which is the homozygous mutant for the MAPKK of the HOG pathway and *hog1*Δ/Δ*pbs2*Δ/Δ cells in the spot assay and observed their variable susceptibility to Tm (Figure 1B). In order to further characterize the differential behavior of *hog1*Δ/Δ, *pbs2*Δ/Δ, and *hog1*Δ/Δ*pbs2*Δ/Δ on Tm, we performed an experiment wherein the kinetics of viability was measured for these mutants in presence of Tm. Cells from exponential phase were treated with a lethal concentration of Tm (20 μg/ml) in liquid YEPD medium and samples were collected at different times and spotted on YEPD plates. As shown in Figure 1C, the *hog1*Δ/Δ mutant lost viability faster than not only the wild type but also the *pbs2*Δ/Δ and *hog1*Δ/Δ*pbs2*Δ/Δ mutants. Furthermore, the *pbs2*Δ/Δ and the *hog1*Δ/Δ*pbs2*Δ/Δ mutants were slow in losing viability, compared to *hog1*Δ/Δ cells (Figure 1C). This set of data indicates that: (i) the ability of Hog1 to counter ER stress may be only partly dependent on the phosphorylating activity of the Pbs2 MAPKK as indicated by the decreased susceptibility of *pbs2*Δ/Δ cells to Tm (Figure 1C), and (ii) decreased susceptibility of the *hog1*Δ/Δ*pbs2*Δ/Δ cells, compared to the parent mutant strains, could be through the activation of alternate pathways consistent with the role of a functional Hog1 in repressing cross-talk with other MAPK pathways (Eisman et al., 2006; Cheetham et al., 2011).

HOG pathway operates via two distinct branches. The first branch consists of the two-component protein, Sln1 and the Ypd1 and Ssk1 phosphorelay system, while the second branch operates through the transmembrane adaptor protein Sho1 (Alonso-Monge et al., 2006). We therefore assessed the contribution of these branches toward the survival of *C. albicans* during ER stress and included the *ssk1*Δ/Δ and *sho1*Δ/Δ mutants in Tm susceptibility assay. The *ssk1*Δ/Δ mutant displayed increased susceptibility to Tm, compared to the *sho1*Δ/Δ mutant (Figure 1B), indicating that Hog1-mediated ER stress resistance may have an increased dependency on the Ssk1 response regulator of the HOG pathway in *C. albicans.* Taken together, this set of data points to the requirement of HOG MAPK pathway during ER stress in *C. albicans.*

### *SSK1* and the Canonical Unfolded Protein Response Pathway Mediate Hog1 Activation During Endoplasmic Reticulum Stress

**FIGURE 2:**
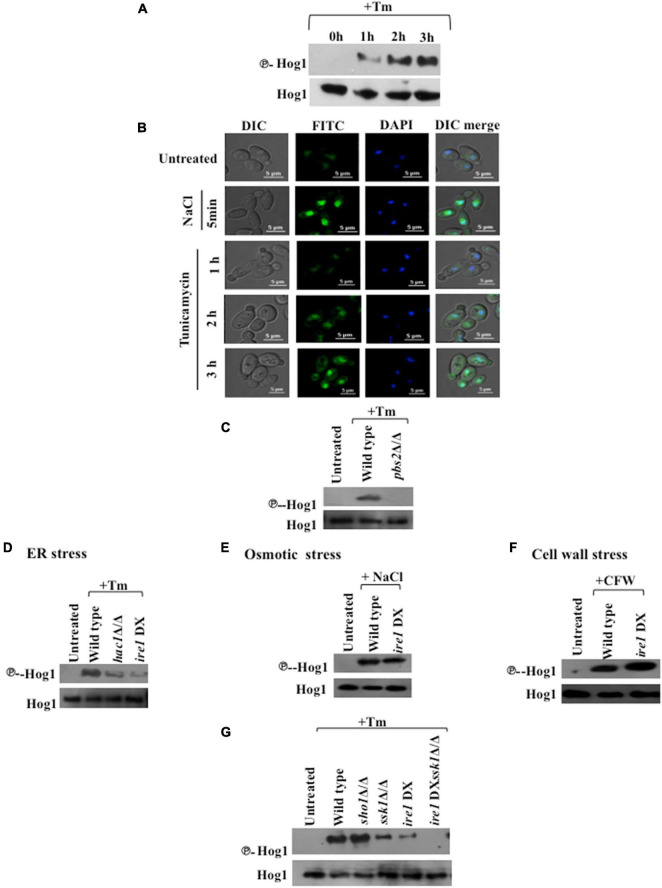
Hog1 phosphorylation during ER stress is dependent on *SSK1* and the UPR. **(A)** Phospho-Hog1 Western blot of wild type strain treated with 2 μg/ml tunicamycin for indicated time periods. **(B)** Cells expressing Hog1-GFP were grown to early log phase at 30°C and treated with 1 M NaCl for 5 min and 2 μg/ml of Tm for indicated time points. The cells were collected and viewed by confocal microscope to monitor Hog1-GFP localization. DAPI (4′,6-diamidino-2-phenylindole) is used for 15 min to stain nucleus. **(C–G)** Phospho-Hog1 Western blots of indicated strains treated with 2 μg/ml tunicamycin for 3 h, 1 M NaCl for 5 min, and 50 μg/ml of calcoflour white for 2 h.

To investigate the involvement of Hog1 in the ER stress response, we examined the phosphorylation status of Hog1 in wild type cells following exposure to Tm (2 μg/ml). Hog1 phosphorylation was induced significantly after 2 h treatment with Tm (Figure 2A), pointing to the contribution of Hog1 during late phase of ER stress in *C. albicans*. To investigate if phosphorylated Hog1 accumulates in the nucleus, we monitored the localization of Hog1-GFP in wild type *C. albicans* cells. For this, the cells were treated with Tm (2 μg/ml) up to 3 h and 1 M NaCl for 5 min, and samples were collected at different time points. Hog1-GFP accumulated in the nucleus rapidly, following exposure to 1M NaCl for 5 min. Contrastingly, while moderate accumulation of Hog1-GFP was detected in the cytosol, it failed to accumulate in the nucleus, following Tm exposure for 1 h (Figure 2B). At time points later than 1 h, Hog1-GFP was detected in the nucleus with a concomitant enrichment of GFP in the cytosol in agreement with its phosphorylation status (Figure 2B). This data suggests that: (i) ER stress-induced Hog1 phosphorylation and its subsequent translocation to the nucleus is a less pronounced and slow process, compared to the response of Hog1 to osmotic stress and (ii) Hog1 may be required specifically during late phase of ER stress in *C. albicans*.

Hog1 is activated by Pbs2 MAPKK-dependent phosphorylation resulting in its migration to the nucleus in response to osmotic stress (Alonso-Monge et al., 2003; Smith et al., 2004; Arana et al., 2005). We therefore examined Hog1 phosphorylation levels in *pbs2*Δ/Δ cells following exposure to Tm (2 μg/ml; 3 h). Hog1 phosphorylation was abrogated in *pbs2*Δ/Δ cells (Figure 2C), indicating that Hog1 is phosphorylated in a Pbs2-dependent manner during ER stress in *C. albicans*.

Considering that Ire1 is the sole sensor of ER stress in fungi (Krishnan and Askew, 2014), we questioned its mode of communication with the HOG pathway during ER stress. To answer this question, Hog1 phosphorylation was checked in *hac1*Δ/Δ and *ire1* DX mutant strain treated with Tm (2 μg/ml; 3 h), two main components of the conserved UPR pathway in this fungus. Both the mutants exhibited significantly reduced levels of phosphorylated Hog1, indicating toward the essentiality of functional UPR pathway for full-blown activation of the HOG pathway during ER stress (Figure 2D).

Given that ER stress is one of the underlying causes for alterations in cell wall integrity (Krysan, 2009; Sircaik et al., 2021), it is possible that activation of Hog1 during ER stress is an indirect effect of cell wall stress. Moreover, Hog1 is also activated by osmotic stress in *C. albicans* (Alonso-Monge et al., 2003), questioning again the direct effect of ER stress on the activation of Hog1. To answer this question, *ire1* DX mutant strain was treated with 1 M NaCl (10 min) and Calcofluor white (50 μg/ml; 2 h) to induce osmotic and cell wall stresses, respectively. While the activation of Hog1 remained unaffected during osmotic stress (Figure 2E), Hog1 phosphorylation in *ire1* DX mutant strain was slightly enhanced in response to the cell wall stressor (Figure 2F), in accordance with previous reports that demonstrate an exacerbated cell wall stress in the absence of functional UPR (Krysan, 2009; Sircaik et al., 2021). This result was in contrast to the significantly reduced Hog1 phosphorylation in *ire1* DX mutant strain during Tm-induced ER stress (Figure 2D). Thus, these results suggest that *C. albicans* utilizes a mechanism, discrete from that of osmotic or cell wall stress, to induce Hog1 phosphorylation during late phase ER stress. Considering that the absence of Ire1 and Hac1 resulted in diminished but not abrogated phosphorylation of Hog1, our result points to the contribution of a functional Ire1 in regulating the activity of the HOG pathway during ER stress.

Given that the HOG pathway is composed of Sho1- and Sln1-dependent branches that are responsive to different stresses, we sought to identify its mediator branch during Tm-induced ER stress. Following exposure to Tm, *sho1*Δ/Δ cells showed wild type levels of Hog1 phosphorylation, in contrast to the significantly reduced phosphorylation of Hog1 in cells lacking *SSK1* (component of Sln1 branch) (Figure 2G). This is suggestive of a prominent role of the Ssk1 response regulator in mediating Tm-induced ER stress in *C. albicans*.

To define the relationship between the Ire1-dependent UPR pathway and the Ssk1-mediated activation of HOG pathway during ER stress, we created a *ire1*DX*ssk1*Δ/Δ (Table 1) double mutant strain. Hog1 phosphorylation was completely eliminated in the double mutant (Figure 2G). Taken together, these results indicate the dependency of Hog1 phosphorylation on a functional Ire1-dependent UPR and the Ssk1 mediator branch during ER stress. All told, we propose that Hog1 phosphorylation during ER stress is not the result of indirect activation of osmotic stress or cell wall stress pathways and UPR and HOG pathways operate in parallel to combat ER stress in *C. albicans*.

### Hog1 Terminates Ire1-Dependent Unfolded Protein Response to Promote Adaptation to Endoplasmic Reticulum Stress

**FIGURE 3:**
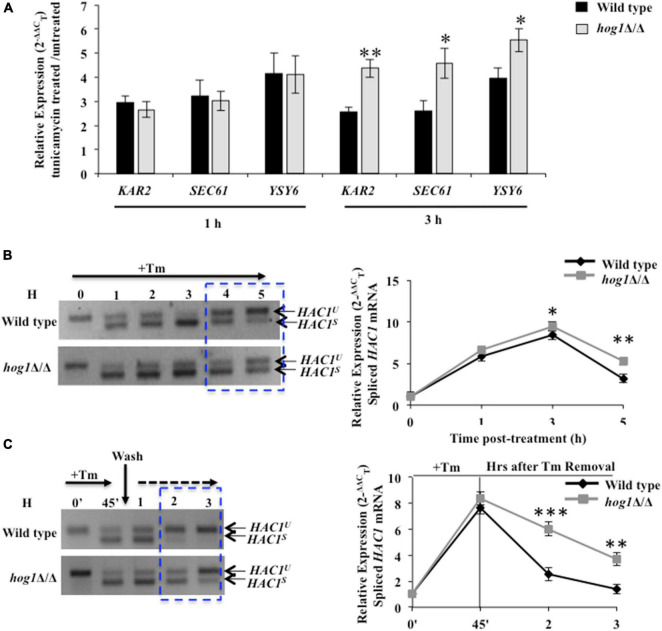
*hog1*Δ/Δ retains high levels of spliced *HAC1* mRNA during ER stress. **(A)** qRT-PCR of *HAC1*-dependent genes in wild type and *hog1*Δ/Δ cells following exposure to 2 μg/ml of tunicamycin for 1 h and 3 h. **(B)** and **(C)** *HAC1* mRNA analysis in wild type and *hog1*Δ/Δ exposed to 2 μg/ml of tunicamycin for indicated time points. Total RNA was isolated from the indicated strains, cDNA was prepared and subject to PCR using *HAC1* specific splicing primers; 4% agarose gel was run to check the PCR products. Graphs depict qRT-PCR with TaqMan probe specific for spliced *HAC1 (HAC1*^s^*)* or unspliced *HAC1* isoform (*HAC1*^u^*)* in wild type and *hog1*Δ/Δ following tunicamycin exposure. Values are the means of at least three independent experiments. The data were analyzed statistically using Student’s *t*-test: *p ≤ 0.05; ^**^p ≤ 0.01; ^***^p ≤ 0.001.

Given the requirement of functional Ire1 for Tm-induced full-blown activation of Hog1, we were prompted to analyze the basis of the cross-talk between the HOG and canonical Ire1-Hac1-dependent UPR pathways. *Candida albicans* depends on the prompt activation of the canonical UPR to abate early waves of ER stress (Sircaik et al., 2021). Subsequently, when the ER function is restored, adaptation to ER stress occurs, and the UPR is terminated. UPR activity is monitored by analyzing the kinetics of appearance and disappearance of the *HAC1^s^* (spliced) mRNA. During late stage of ER stress, the disappearance of the *HAC1^s^* mRNA reflects the timely termination of the UPR activity that is shown to correlate with recovery of ER functions and, thus, adaptation to ER stress in *S. cerevisiae* (Chawla et al., 2011). Therefore, to test for a relationship between HOG and Ire1-Hac1-dependent UPR pathways, we traced Ire1 activity by monitoring the processing of *HAC1^u^* (unspliced) mRNA and the transcriptional induction of the Hac1-dependent genes (*KAR2, SEC61*, and *YSY6*) in the wild type and *hog1*Δ/Δ cells (Wimalasena et al., 2008; Thomas et al., 2015; Sircaik et al., 2021). We monitored the induction of the aforesaid genes, following exposure of wild type and *hog1*Δ/Δ cells to Tm for 1 h and 3 h, as phosphorylation of Hog1 was also examined at these same time points (Figure 2A). Our analysis shows that gene expression in *hog1*Δ/Δ cells remain unchanged relative to the wild type following Tm exposure for 1 h (Figure 3A). However, at 3 h, the transcript levels of *KAR2, SEC61*, and *YSY6* were 1.7–, 1.7–, and 1.4-fold upregulated, respectively, in the mutant compared to the wild type (Figure 3A). We hypothesized that the increased expression of the Hac1-dependent genes in *hog1*Δ/Δ cells may be due to an alteration in the kinetics of UPR activation. To this end, the kinetics of appearance of the *HAC1^s^* (spliced) mRNA in Tm (2 μg/ml) treated wild type and *hog1*Δ/Δ cells was examined by (a) performing RT-PCR using primers across the *HAC1* mRNA intron followed by analyzing the PCR products on agarose gel and (b) using Taqman probes for higher efficiency during target amplification. The *HAC1^s^* mRNA levels appeared in both the wild type and the mutant within 1 h of Tm treatment as inferred from examining the PCR products on agarose gel (Figure 3B). This observation resonated with the 6-fold increase in the *HAC1^s^* mRNA levels within 1 h of Tm treatment, measured by the Taqman probe analysis in wild type as well as the mutant (Figure 3B). Consistently, transcriptional induction of Hac1-dependent genes occurred to similar levels in both the wild type and the mutant (Figure 3A) at 1 h. After 3 h, there was a decrease in the level of *HAC1^s^* mRNA with a concurrent increase in the *HAC1^u^* mRNA levels in the wild type (Figure 3B). In contrast, *hog1*Δ/Δ cells exhibited increased *HAC1^s^* mRNA and decreased *HAC1^u^* mRNA at the 4 h and 5 h time points (Figure 3B). In agreement with this observation, Taqman probe analysis demonstrated the presence of 2-fold elevated levels of *HAC1^s^* mRNA in the *hog1*Δ/Δ cells, compared to the wild type at 5 h (Figure 3B). This result suggests that while the decline of the *HAC1^s^* mRNA levels in the wild type was rapid, *hog1*Δ/Δ cells retained the *HAC1^s^* mRNA levels for a longer duration. This observation correlated well with the increased transcriptional induction of the Hac1-dependent genes following Tm exposure for 3 h, compared to the wild type (Figure 3A). The slow kinetics of disappearance of *HAC1^s^* mRNA in the mutant may reflect delayed termination of the UPR and adaptation to ER stress.

To further test the correlation between the disappearance of *HAC1^s^* mRNA and adaptation to ER stress, we performed UPR recovery experiments. We presumed that if disappearance of *HAC1^s^* mRNA occurs due to attenuation of UPR, then removing the stressor (Tm) followed by growth of the wild type in medium lacking Tm should enhance the disappearance of *HAC1^s^* mRNA. However, if the absence of Hog1 affects the timely attenuation of UPR, then the kinetics of disappearance of *HAC1^s^* mRNA should be slower in the mutant. To this end, strains of interest were treated with Tm for 45 min followed by their transfer to fresh growth medium lacking Tm. At 3 h after Tm washout, spliced *HAC1* mRNA levels declined by 8-fold in the wild type, compared to its levels at the start of the washout (45 min, following Tm exposure) (Figure 3C). The rapid decline was well reflected in Lane 5 of Figure 3C as spliced *HAC1* mRNA levels were mostly undetectable at 3 h, thus forging a link between the rapid disappearance of spliced *HAC1* mRNA with the timely attenuation of the UPR. In contrast, *hog1*Δ/Δ cells exhibited only a 2-fold decline in the spliced *HAC1* mRNA levels at 3 h after Tm washout, compared to the wild type (Figure 3C). This observation was in agreement with the RT-PCR result, providing further support to our hypothesis that the slow disappearance of *HAC1^s^* mRNA in the absence of Hog1 reflects late attenuation and thus, recovery from ER stress in *C. albicans*. Altogether, we conclude that during ER stress: (i) the activity of the HOG pathway modulates the UPR pathway such that the absence of functional Hog1 causes sustained activation of the latter; and (ii) the role of Hog1 during ER stress could be tied to its ability to promptly terminate Ire1-dependent UPR for restating ER homeostasis in *C. albicans*.

### Hog1 Influences Adaptation to Endoplasmic Reticulum Stress by Regulating the Basal Levels of Glycerol in *Candida albicans*

**FIGURE 4:**
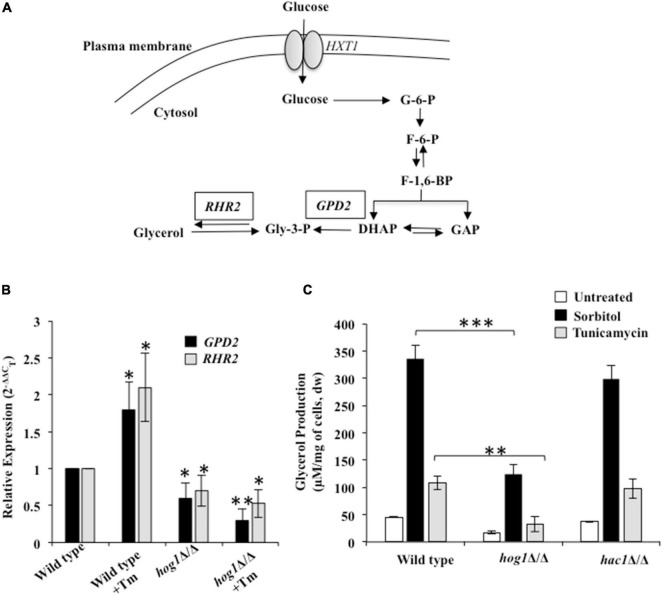
Hog1-dependent glycerol production during ER stress. **(A)** Glycerol synthesis pathway in *C. albicans.* HXT: hexose transferase; G-6-P: Glucose-6-phosphate; F-6-P: Fructose-6-phospate; F-1, 6-BP: Fructose-1,6-biphosphate; DHAP: Dihydroxyacetone phosphate; GAP: Glyceraldehyde-3-phosphate; Gly-3-P: Glycerol-3-phosphate; *RHR2*: glycerol-3-phosphatase; *GPD2*: glycerol-3-phosphate dehydrogenase. **(B)** qRT-PCR to measure transcript levels of *GPD2* and *RHR2* following treatment 2 μg/ml of tunicamycin for 3 h. **(C)** Wild type and indicated mutant strains were grown in YEPD at 30°C and then transferred to fresh medium containing 1 M sorbitol or 2 μg/ml of tunicamycin. After 3 h, aliquots (1 ml) of the cultures were withdrawn and processed for glycerol content. Values represent the difference in total glycerol before and after the indicated treatment and are the means of at least three independent experiments. dw, dry weight. The data was analyzed statistically using Student’s *t*-test: *p ≤ 0.05; ^**^p ≤ 0.01; ^***^p ≤ 0.001.

Prior studies in *S. cerevisiae* show that the protective effect of the HOG pathway during osmotic and ER stresses can be partially attributed to its cytosol-dependent role in regulating glycerol production. Glycerol is a natural osmolyte shown to protect cellular proteins from aggregation, during hostile conditions (Albertyn et al., 1994; Hohmann, 2002; Kumar, 2009; Torres-Quiroz et al., 2010; Petelenz-Kurdziel et al., 2013). Endoplasmic reticulum stress-induced accumulation of misfolded proteins activate *GPD1* (glycerol-3-phosphate dehydrogenase) and *GPP1*/*RHR2* (glycerol-3-phosphatase) genes required to produce glycerol (Figure 4A) in a Hog1-dependent manner in *S*. *cerevisiae* (Albertyn et al., 1994; Hohmann, 2002; Enjalbert et al., 2006). In *C. albicans*, while this MAPK induces *GPD2* (isozyme of *GPD1*) and *RHR2*-mediated glycerol production during osmotic stress, their role during Tm-induced ER stress remains uninvestigated (Day et al., 2017). Considering this background, we hypothesized that aside from assisting the termination of the Ire1-dependent UPR pathway, Hog1 may also be influencing adaptation to ER stress through its ability to induce glycerol production in *C. albicans*. Functional Hog1 is required for regulating basal levels of both *GPD2* and *RHR2* as evidenced by their reduced transcript levels in untreated *hog1*Δ/Δ cells, compared to the wild type. Following Tm exposure, while the wild type exhibited 2-fold increase in the transcript levels of *GPD2* and *RHR2*, the transcript level of these genes was significantly downregulated in *hog1*Δ/Δ cells (Figure 4B). This data points to the essentiality of functional Hog1 in regulating the transcription of *GPD2* and *RHR2* in basal as well as Tm-induced ER stress conditions in *C. albicans*.

To correlate the reduced transcript levels of the aforesaid genes with glycerol production, we measured glycerol levels in wild type, *hog1*Δ/Δ, and *hac1*Δ/Δ cells exposed to Tm and sorbitol. Considering that glycerol production in *S. cerevisiae* occurs in response to osmostress (Torres-Quiroz et al., 2010), we included wild type cells exposed to 1 M sorbitol as a control for the experiment. In the absence of Tm, the intracellular glycerol content was reduced by 3-fold in *hog1*Δ/Δ cells, compared to the wild type. Despite the downregulated expression of *GPD2* and *RHR2*, when *hog1*Δ/Δ was exposed to Tm or sorbitol, the intracellular glycerol content increased by 2- and 7-fold, respectively, compared to the untreated mutant strain (Figure 4C). These fold increases in the glycerol content were similar to that observed with the wild type exposed to Tm or sorbitol. This data points to the role of Hog1 in regulating the basal levels of glycerol, which coupled with the reduced transcript levels of *GPD2* and *RHR2* contributes to lower glycerol levels in the mutant in absence of the ER stressor. Moreover, it is plausible that ER stress-induced glycerol production may only be partially dependent on a functional Hog1 and the possibility of glycerol production in Hog1-independent manner cannot be ruled out in *C. albicans* (Figure 4C). Interestingly, *hac1*Δ/Δ cells exhibited glycerol content similar to the wild type, indicating absence of functional interaction between the HOG and the UPR pathways for glycerol production. Given the increased susceptibility of *hog1*Δ/Δ cells to Tm (Figure 1), it is likely that Hog1, by regulating basal levels of glycerol, may be involved in conferring protection to *C. albicans* during ER stress.

### Cytosolic as Well as Nuclear Activity of Hog1 Is Required for Mediating Endoplasmic Reticulum Stress Resistance

**FIGURE 5:**
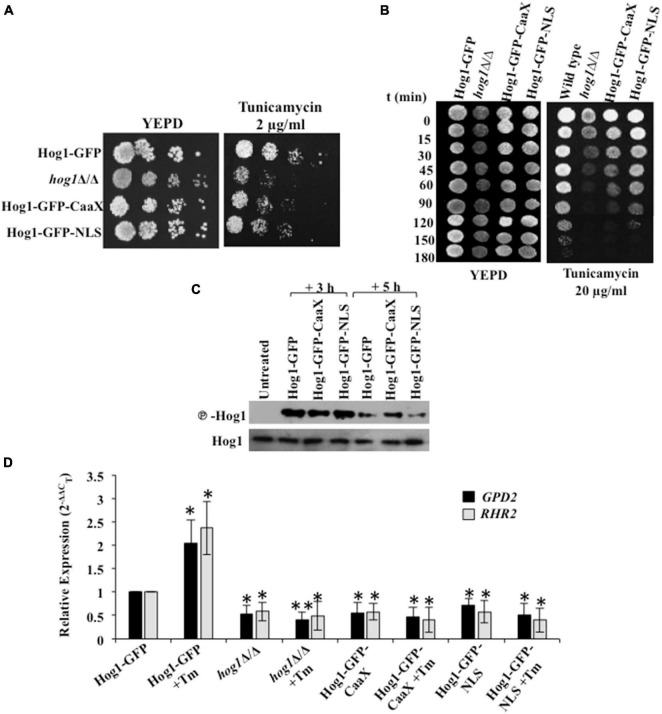
Hog1 depends on its cytosolic and nuclear activity for ER stress resistance. **(A)** Serial dilutions of cell suspensions of the indicated strains were spotted onto YEPD plates supplemented with 2 μg/ml of tunicamycin, and incubated at 30°C for 48 h. **(B)** Exponentially growing cells of indicated strains were exposed to 20 μg/ml tunicamycin in liquid YEPD at 30°C. Samples were taken at indicated time points and spotted onto YEPD plates (10^5^ cells in 10 μl) and incubated for 24 h at 30°C. **(C)** Phospho-Hog1 Western blot of indicated strains treated with 2 μg/ml tunicamycin for 3 h and 5 h. **(D)** qRT-PCR of *HOG1*-dependent target genes (*GPD2* and *RHR2*) upon treatment with 2 μg/ml of tunicamycin for 3 h. The data was analyzed statistically using Student’s *t*-test: *p ≤ 0.05; ^**^p ≤ 0.01.

Preventing nuclear accumulation of *C. albicans* Hog1 does not impair the activation of Hog1-dependent osmo-protective genes (Day et al., 2017). Thus in this fungal pathogen, nuclear accumulation of Hog1 is not essential for the transcriptional regulation of osmo-responsive genes. To examine the contribution of the cytosolic and nuclear activity toward Hog1-mediated ER stress resistance, we used strains that consisted of Hog1 that was either tethered to the plasma membrane (Hog1-GFP-CaaX) or constitutively nuclear (Hog1-GFP-NLS) (Day et al., 2017). While both the strains were partially able to resist ER stress, the Hog1-GFP-NLS cells conferred increased stress resistance, compared to the Hog1-GFP-CaaX carrying cells (Figures 5A,B). The inability of the Hog1-GFP-NLS cells to exhibit wild type levels of resistance to Tm pointed to the partial dependency of Hog1 on its cytosolic activity for mounting an effective ER stress response.

Because cells in which Hog1 is plasma membrane tethered retained their ability to partially resist ER stress, we speculated that the protective effect of Hog1-GFP-CaaX could be through: (i) influencing the activation of Hog1, (ii) the induction of the ER stress-specific transcriptional program, or (iii) its influence on promoting recovery from ER stress. As phosphorylation of Hog1 is a prerequisite for mediating its activity, we first sought to examine the impact of Hog1 cellular localization on its phosphorylation status following Tm exposure for 3 h and 5 h. Phosphorylation of the Hog1-GFP-CaaX fusion lasted 2 h longer than the wild type and the Hog1-GFP-NLS fusion (Figure 5C). Thus, activation of Hog1 in response to Tm-induced ER stress is not dependent on its cellular localization. This suggests that the partial ER stress resistance exhibited by *C. albicans* cells expressing Hog1-GFP-CaaX could be due to increased Hog1 phosphorylation.

Second, presuming that nuclear accumulation is not essential for inducing ER stress-responsive gene expression in *C. albicans*, we measured the transcript levels of *GPD2* and *RHR2* given their Hog1-dependent expression following Tm exposure (Figure 4B). Interestingly, while the expression of *GPD2* and *RHR2* was induced ≥1.5-fold in the wild type, their expression was abolished in the cells expressing GFP-CaaX fusion protein treated with Tm for 3 h, correlating well with the expression profile in *hog1*Δ/Δ cells (Figure 5D). This observation shows that partial resistance of Hog1-GFP-CaaX to ER stress is not fully dependent on its nuclear translocation and the subsequent triggering of the transcriptional program. Striking was a similar abrogation of *GPD2* and *RHR2* in cells expressing the GFP-NLS fusion protein, suggestive of the dependency of the nuclear localized Hog1 on the expression of Hog1-dependent cytosolic proteins for transcriptional regulation.

**FIGURE 6:**
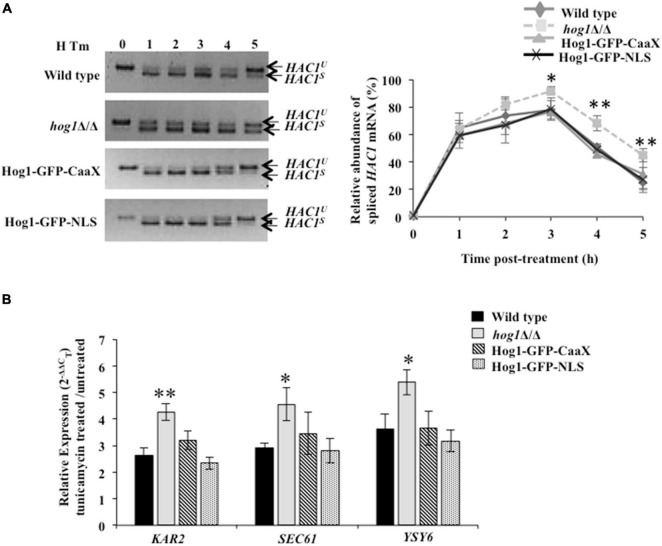
Hog1 is dependent on both cytosolic and nuclear activity for promoting recovery from ER stress. **(A)** *HAC1* mRNA analysis in indicated strains treated with 2 μg/ml of tunicamycin for the indicated time. Total RNA was isolated from the indicated strains, cDNA was prepared and 4% agarose gel was run to check for PCR products. Graph depicts relative abundance of spliced *HAC1* mRNA (%). Values of relative abundance of spliced *HAC1* mRNA were calculated by using the formula: (band intensity of *HAC1^s^* mRNA)/[(band intensity of *HAC1^s^* mRNA) + (band intensity of *HAC1^u^* mRNA)]. **(B)** qRT-PCR of *HAC1*-dependent UPR target genes in indicated cells upon treatment with 2 μg/ml of tunicamycin for 3 h. The data were analyzed statistically using Student’s *t*-test: *p ≤ 0.05; **p ≤ 0.01.

Third, considering the absence of transcriptional response in *C. albicans* cells carrying Hog1-GFP-CaaX, we asked what other cellular process does Hog1 impact to resolve ER stress. We hypothesized that Hog1-GFP-CaaX fusion protein may be mediating ER stress resistance by promoting the timely attenuation of Ire1-dependent UPR. To address this, processing of *HAC1^u^* mRNA and the subsequent activation of the UPR genes was monitored. While cells lacking *HOG1* exhibited a 30% decline in *HAC1^s^* mRNA levels (Figure 6A; closed squares), the wild type, Hog1-GFP-CaaX, and Hog1-GFP-NLS cells show 60% decline in *HAC1^s^* mRNA levels, pointing to the slow processing of *HAC1^u^* mRNA in *hog1*Δ/Δ cells (Figure 6A). Consequentially, both the fusion proteins displayed wild type levels of inductions of the Hac1-dependent genes in Tm-treated cells for 3 h (Figure 6B). This indicated that *C. albicans*’ ability to attenuate the UPR activity and promote recovery from ER stress is not impacted by its cellular localization, an attribute that may underly the partial ER stress resistance exhibited by both GFP-CaaX and -NLS cells.

Taken together, we surmise that during ER stress (i) the ability of Hog1 to activate ER stress-responsive genes may be dependent on the induction of a significant group of additional factors that require the cytosolic activity of this transcription factor, (ii) nuclear accumulation of Hog1 is not a pre-requisite for terminating the Ire1-dependent UPR activity and promoting recovery from ER stress, and (iii) cytosolic as well as nuclear activity of Hog1 are essential for mediating its protective effect during ER stress in *C. albicans*.

## Discussion

Endoplasmic reticulum (ER) stress response pathways and their regulation have a profound effect on the pathogenicity of *C. albicans*. The fungus relies on the activation of the Ire1-dependent UPR pathway for countering ER stress, an attribute that facilitates the pathogenicity of this fungus (Sircaik et al., 2021). Subsequent sequence of events leading to the attenuation of the UPR and restoration of ER homeostasis remain an undetermined facet of the ER stress response in *C. albicans*. The involvement of the HOG MAPK pathway in countering multiple stresses is well established in *C. albicans* (Román et al., 2020). Herein, we elucidate the position of the HOG MAPK pathway within the little known framework of UPR in *C. albicans*. On the basis of the increased susceptibility of cells lacking components of the HOG MAPK pathway to Tm, we inferred the involvement of this pathway in protection against ER stress in *C. albicans* (Figure 1). Furthermore, we propose a role of this MAPK during the late phase of ER stress in the fungus as phosphorylation of Hog1 and its nuclear enrichment was elevated following Tm exposure for extended length of time (up to 3 h) (Figures 2A,B).

**FIGURE 7:**
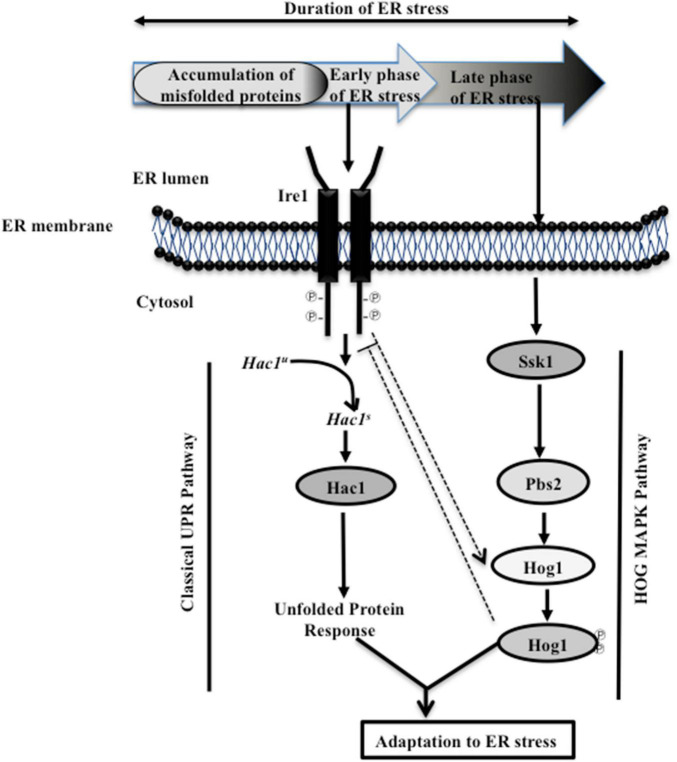
Model depicting cellular responses during ER stress. Early (initial) exposure to ER stress initiates the autophosphorylation of Ire1, leading to the activation of its ribonuclease (RNase) activity and the subsequent splicing of the intron from *HAC1^u^* (unspliced) mRNA. Spliced (*HAC1^s^*) mRNA codes for the Hac1 protein, which activates the UPR target genes. During ER stress, Ire1 promotes the activation of HOG MAP kinase pathway (indicated by dotted arrow). Subsequently, adaptation to ER stress is allowed, when Ssk1-mediated activation of Hog1 MAP kinase attenuates the activity of Ire1 (indicated by dotted blunt line). The attenuation of Ire1 activity could be due to the influence of Hog1, directly or indirectly, on the dephosphorylation of Ire1, which in turn regulates the RNase activity-mediated processing of *HAC1^u^* mRNA. As the UPR is terminated, the amplitude of the signal through the HOG pathway decreases and the stress response is fully terminated. Consequentially, the concerted action of the UPR and HOG MAPK pathways facilitate adaptation to ER stress, and guides the recovery of *C. albicans* from ER stress.

The results presented here uncover a functional relationship between HOG MAPK and the Ire1-dependent UPR pathways to show that parallel working of these signaling pathways is essential for adaptation and thus recovery of *C. albicans* from ER stress (Figure 7). During the initial phase of ER stress, after protein homeostasis is restored via the Ire1-Hac1-dependent pathway, the UPR has to subside. The ribonuclease (RNase) activity-dependent processing of *HAC1* mRNA is initiated and terminated by autophosphorylation followed by dephosphorylation of Ire1, respectively. Consequentially, at the later stage of ER stress, the dephosphorylation event suppresses the RNase activity, thus reducing the level of spliced *HAC1* mRNA and terminating the UPR in *S. cerevisiae*. In agreement, cells carrying mutations in the kinase domain of Ire1 exhibited elevated level of spliced *HAC1* mRNA and increased susceptibility to ER stressor in the yeast (Chawla et al., 2011; Chang et al., 2018). Considering this background, we attributed the inability of *C. albicans hog1*Δ/Δ cells to sustain growth in the presence of Tm to its role in supporting UPR attenuation (Figure 1). This explanation is also in line with the activation of the HOG pathway solely during the late stage of ER stress (Figure 2A). Decreased levels of the spliced *HAC1* mRNA after 5 h of Tm treatment indicated recovery from ER stress and, thus, termination of the UPR in the wild type (Figure 3B). Contrastingly, the continuous expression of spliced *HAC1* mRNA (*HAC1^s^*) even after the removal of the ER stressor (Figure 3C) suggested delayed termination of the UPR in *hog1*Δ/Δ cells. The influence of Hog1 in attenuating the Ire1-dependent UPR can be explained by a possible direct or indirect involvement of the MAP kinase in promoting the dephosphorylation and, thus, inactivation of Ire1, when challenged with ER stress. As a consequence, the processing of *HAC1* mRNA will cease resulting in the downregulation of Hac1-dependent genes, thus enabling adaptation to ER stress. As the attenuation of the Ire1-dependent UPR occurs, the amplitude of the signal through the HOG pathway may also decrease and the stress response is fully terminated (Figure 7). In agreement, compromising the expression of Ire1 (*ire1* DX mutant strain) reduced the levels of phosphorylated Hog1 following Tm exposure for extended times (Figures 2B,G). One plausible explanation for this observation could be that Ire1 downregulates the expression of Hog1-specific phosphatases, thus allowing *C. albicans* to maintain normal levels of phosphorylated Hog1. Alternatively, Ire1-dependent transcriptional programming may also involve upregulating genes that facilitate Hog1 phosphorylation, aspects of ER stress that can be explored in future. We propose that by virtue of the involvement of HOG pathway in the timely attenuation of the UPR activity, the unnecessary activation of the Ire1-dependent UPR pathway is impeded, once ER homeostasis is restated, in this pathogenic fungus. Regulating the activity of Ire1-dependent UPR as a mode to modulate the ER stress response also occurs through Snf1 in *S. cerevisiae*. Snf1 not only downregulates the expression of Ssk1 to influence the activity of the HOG pathway but also inactivates the UPR in *S. cerevisiae* (Mizuno et al., 2015). This points to the existence of multiple modes of inactivating the UPR, albeit the unexplored role of Snf1 during ER stress in *C. albicans*.

The diversity in the upstream components that activate Hog1, the magnitude or duration of Hog1 phosphorylation, and a difference in the localization pattern of this MAPK in response to various stresses influences the specificity of the downstream events (Román et al., 2020). *C. albicans* deploys Sln1 two-component protein for the activation of Hog1 during osmotic and oxidative stresses (Chauhan et al., 2003; Alonso-Monge et al., 2006; Cheetham et al., 2007). We show that during ER stress, both functional Ssk1 and Ire1 are essential for Hog1 phosphorylation and thus its activation (Figure 2G). Considering that ER and oxidative stress co-exist in yeast (Guerro-Moreno et al., 2019), it is possible that oxidative stress was the basis of Hog1 activation during prolonged ER stress. This possibility was partly ruled out as the absence of Ssk1 is shown to abolish oxidative stress-induced phosphorylation of Hog1 in *C. albicans* (Chauhan et al., 2003). Alternately, it is plausible that activation of anti-oxidative stress responses that are Ssk1-independent facilitate protection from oxidative stress during ER stress and that it is through this combined action of anti-ER and -oxidative stress responses that facilitate *C. albicans* adaptation to ER stress. If ER and oxidative stress co-exist and whether the latter modulates ER stress response in Ssk1-dependent or -independent manner are features of the UPR that remain unexplored in *C. albicans*. Despite the similarity in the upstream components, ER stress induced both nuclear and cytoplasmic localization of Hog1 (Figure 2B), unlike its exclusive localization in the nucleus during osmo- and oxidative stresses, respectively (Smith et al., 2004; Arana et al., 2005). This was suggestive of a role for Hog1 in regulating responses to ER stress by functioning from both cytoplasm and nucleus. This observation is evidenced as follows: (i) both the cytosolic and nuclear localized Hog1 were partially able to resist growth in presence of Tm (Figures 5A,B) and (ii) nuclear enriched Hog1 (Hog1-NLS-GFP) exhibited abrogated expression of Hog1-dependent ER stress-responsive genes, *GPD2* and *RHR2* (Figure 5D). It is plausible that Hog1-dependent cytosolic targets may migrate to the nucleus for activating ER stress-responsive transcriptional program. Contrary to this observation, Hog1 does not depend on nuclear localization for expression of osmo-responsive genes in *C. albicans*, challenging the essentiality of nuclear translocation for inducing Hog1-dependent gene expression in response to all stresses (Day et al., 2017). Moreover, during ER stress, the cells expressing Hog1-CaaX-GFP retained phosphorylation for longer duration (Figure 5C), similar to osmotic stress conditions but in contrast to oxidative stress signal (Day et al., 2017). Dephosphorylation of Hog1 in *S. cerevisiae* is ascribed to the nuclear localized tyrosine phosphatase Ptp2 (ScPtp2) (Wurgler-Murphy et al., 1997). Likewise, the sustained phosphorylation of Hog1-GFP-CaaX can be attributed to its inaccessibility to the presumably nuclear localized phosphatases Ptp2 and Ptp3 (orthologs of ScPtp2) (Su et al., 2013). Our findings reaffirm the existence of stress-specific unique mechanisms that differentially affect the activation and cellular localization of Hog1, thus imparting specificity to the downstream outcomes in *C. albicans*.

Interestingly, a functional Hog1 is sufficient to promote adaptation to ER stress as both cytosolic as well as nuclear localized Hog1 was able to terminate Ire1-dependent UPR independently (Figure 6A). We speculate that Hog1 may be responding to ER stress in a biphasic manner, where the modification of preexisting cytosolic proteins may initiate the desensitization and termination of Ire1-dependent UPR, followed by the induction of Hog1-dependent nuclear genes required for the termination. Thus, this MAPK by virtue of its cytosol-dependent activity provides a fast and direct stress response that facilitates rapid relief from ER stress. Analogous to this observation is the role of Hog1 in regulating osmostress in a sequential manner by influencing the activity of a plasma membrane localized ion transporter during the early stages and subsequent activation of gene expression in *S. cerevisiae* (Proft and Struhl, 2004). Our results show a role of Hog1 in modulating the basal level (uninduced) of glycerol production (Figures 4B,C), a process that may be dependent on the cytosolic activity of Hog1. We show that absence of functional Hog1 is sufficient to reduce the basal glycerol content by 3-fold, compared to wild type (Figure 4C), pointing to a link between the cytosolic activity of Hog1 and glycerol production in *C. albicans*. The nautral osmolyte glycerol is known to shift the folding equilibrium toward the native conformation of the proteins without causing disturbances in other cellular processes, a strategy that allows cells to survive stress created by accumulation of misfolded proteins (Kumar, 2009). Thus, we propose that one of the cytosol-dependent physiological roles of Hog1 could be to induce glycerol production in an attempt to survive ER stress conditions in *C. albicans*. Consistent with this, the role of cytosolic Hog1 in activating the upper branch of the glycolytic pathway, resulting in increased glycerol production during osmotic and ER stress conditions, has been established in *S. cerevisiae* (Torres-Quiroz et al., 2010). However, further work is required to confirm the cytosolic role of *C. albicans* Hog1 for glycerol production during ER stress. Thus, in an attempt to adapt to ER stress and avert cell death, *C. albicans* not only relies on Hac1-dependent structured activation but also on Hog1-dependent organized termination of the canonical Ire1-dependent UPR. Additionally, through this study, the relevance of HOG pathway in fungal pathogenicity can be expanded also to its role in ER stress resistance. The finding that inactivation of HOG MAPK pathway can lock the Ire1-dependent UPR in an activated state for extended duration provides a basis for augmenting cell death in *C. albicans*, an additional vulnerable point for therapeutic intervention.

## Data Availability Statement

The original contributions presented in the study are included in the article/Supplementary Material, further inquiries can be directed to the corresponding author.

## Author Contributions

SP and FH provided the concept and design of the study. FH, PP, and ER performed the experiments and analyzed the data. SP wrote the manuscript. JP and ER reviewed the first draft of the manuscript. All authors approved the submitted version.

## Conflict of Interest

The authors declare that the research was conducted in the absence of any commercial or financial relationships that could be construed as a potential conflict of interest.

## Publisher’s Note

All claims expressed in this article are solely those of the authors and do not necessarily represent those of their affiliated organizations, or those of the publisher, the editors and the reviewers. Any product that may be evaluated in this article, or claim that may be made by its manufacturer, is not guaranteed or endorsed by the publisher.
