# Supplementary material for: Adaptation to Endoplasmic Reticulum Stress in Candida albicans Relies on the Activity of the Hog1 Mitogen-Activated Protein Kinase
Source: Front Microbiol. 2022 Jan 6;12:794855. doi: 10.3389/fmicb.2021.794855 (PMC8770855; doi:10.3389/fmicb.2021.794855)
Supplement: Supplementary file 1 [file Table_1.pdf]

Table S1. Primers used in this study.

Primers	Sequence 5´- 3´
CaCas9/for	ATCTCATTAGATTTGGAACTTGTGGGTT
CaCas9/rev	TTCGAGCGTCCCAAAACCTTCT
SNR52/F	AAGAAAGAAAGAAAACCAGGAGTGAA
SNR/R_SSK1	CCACGTTTATACTTGTTCTGCAAATTAAAAATAGTTTACGCAAGTC
sgRNA/F_SSK1	CAGAACAAGTATAAACGTGGGTTTTAGAGCTAGAAATAGCAAGTTAAA
sgRNA/R	ACAAATATTTAAACTCGGGACCTGG
SNR52/N	GCGGCCGCAAGTGATTAGACT
sgRNA/N	GCAGCTCAGTGATTAAGAGTAAAGATGG
SSK1_del_F	GGGGTTTTGCATAAATACTAATACAAGTTAAACTTTGCTTTAGTGCTCACTTTCTCATACTCAACAGTTCTAGGGGAACCCGTCAAAACTAGAGAATAATAAAGAAAACG
SSK1_del_R	TCATTAAAAGCAAAAACTGAAAAAAACCGAAAACCTAATTTATTCCAACGACTCATCTTAGTGGCATTTCATAAATCCGTGCAGGACCACCTTTGATTGTAAATAG
Comp_SSK1_del_F	CACGTATAAAACTAGACCTCAAGTCTCG
Comp_SSK1_del_R	GATACAGTAAACCTTCCCACCAACC
Up-comp-SSK1	CGTGGAGGATTGGATAATACGC
