# Supplementary material for: Adaptation to Endoplasmic Reticulum Stress in Candida albicans Relies on the Activity of the Hog1 Mitogen-Activated Protein Kinase
Source: Front Microbiol. 2022 Jan 6;12:794855. doi: 10.3389/fmicb.2021.794855 (PMC8770855; doi:10.3389/fmicb.2021.794855)
Supplement: Supplementary file 2 [file Table_2.pdf]

Table S2. Primers used for qRT-PCR in this study.

RHR2/QRT/SLP-F	5'-TGGTCACCAGAAGATGCAATTG-3'
RHR2/QRT/SLP-R	5'-GCCAAAAGTGTCAGGAATAGCA-3'
GPD2/QRT/SLP-F	5'-TGCCTGTTCTGTTGGTTTCGT-3'
GPD2/QRT/SLP-R	5'-TCATAATAGCAGCTTTAGCATTGTCA-3'
ACT1/QRT/SLP-F	5’-GAAGCCCAATCCAAAAGA-3’
ACT1/QRT/SLP-R	5’-CTTCTGGAGCAACTCTCAATTC-3’
SEC61/QRT/SLP-F	5'-GTCACAGAGACACTTCTGCTTACAA-3'
SEC61/QRT/SLP-R	5'-TAGACGTACCAGAACCAAGAGTACC-3'
YSY6/QRT/SLP-F	5'-ACACCTAAACAAAGAGCAGCTAATG-3'
YSY6/QRT/SLP-R	5'-TTGCTCCACCACATACTAAGAA-3'
KAR2/QRT/SLP-F	5'CTGAAGATTACCTTGGCAAAAAAGT-3'
KAR2/QRT/SLP-R	5' -TTAGTAGCTTGTCTTTGAGCATCGTT-3'
SLP/HAC1 SP-F	5’-AGACGCTTTTAATTACCACACCA-3’
SLP/HAC1 SP-R	5’-TCAAAGTCCAACTGAAATG-3’
